# Donation Parties

**Published:** 1856-06

**Authors:** 


### DONATION PARTIES

Are an invention of——the Father of Lies. In our view, Pandora's box could not exhibit a greater variety of ills and concentrated deceptions, hypocrisies and shuffling managements. Just look at it.

You have agreed to pay your minister a certain salary. If that salary is paid, and is fully sufficient for all his purposes, then he does not need any more. If you have so much that you cannot avoid giving, then bestow it on some poor widow, some orphan child in want, or some perishing invalid whom you are sure to have no favors to ask of, and from whom you cannot reasonably expect any reciprocities.

If you are conscious that your minister's salary is not fully adequate to his wants, then stand frankly up to the mark and increase it, thus letting him feel that it is his due, that he can claim it as a right, and that he is not beholden to you in the least. As it is, the Donation system puts him in a false position in several ways.

1. You place him under obligations to you by gifts.

2. You lower him in his own estimation, and in your own, by making him, in some sort, the recipient of your charity.

3. Persons more conscientious and less able than yourself, "*are expected*" to be at the party, when they have already put in as far as they felt themselves able; while others, not willing to be behindhand, have strained themselves more than they would have done had they been left wholly to themselves.

4. Besides these, there is another ill result; persons more considerate than the commonalty, may feel that the salary is not a sufficient one, but then it occurs to them the *annual donation* will make it up, whereas the intrinsic value of those gifts are overrated nine times out of ten, and quite as often, perhaps, are fancy articles of nominal value thrown in, which from their expensiveness, the minister's family may feel inappropriate for their uses, and they are laid by as "keepsakes," totally unavailable for practical purposes.

Be a man, reader, and don't attempt to keep your minister under your thumb in any of these circumbendibus ways; his palms should be as sacred from a bribe as those of the highest judge in the land, he should be as far above the influences of your fear, favor or affection, as a liberal and all-sufficient salary can place him, and feeling this, he can work with a will, and willing hands give a glad heart and a sturdy spirit, which bring in their train high health, a clear head, sound logic and a safe divinity.

